# Xpert MTB/RIF Testing of Stool Samples for the Diagnosis of Pulmonary Tuberculosis in Children

**DOI:** 10.1093/cid/cit230

**Published:** 2013-04-11

**Authors:** Mark P. Nicol, Karalene Spiers, Lesley Workman, Washiefa Isaacs, Jacinta Munro, Faye Black, Widaad Zemanay, Heather J. Zar

**Affiliations:** 1 Division of Medical Microbiology and Institute for Infectious Diseases and Molecular Medicine, University of Cape Town, and National Health Laboratory Service of South Africa, South Africa; 2 Division of Infection and Immunity, University College London, United Kingdom; 3 Department of Paediatrics and Child Health, University of Cape Town; 4 Red Cross WarMemorial Children's Hospital, Cape Town, South Africa

**Keywords:** tuberculosis, children, Xpert, stool, diagnosis

## Abstract

In a pilot accuracy study, stool Xpert testing from 115 children with suspected pulmonary tuberculosis (PTB) detected 8/17 (47%) culture-confirmed tuberculosis cases, including 4/5 (80%) HIV-infected and 4/12 (33%) HIV-uninfected children. Sputum Xpert detected 11/17 (65%) cases. Stool holds promise for PTB diagnosis in HIV-infected children.

## Abstract (precis)

In a pilot accuracy study, stool Xpert testing from 115 children with suspected pulmonary tuberculosis (PTB) detected 8/17 (47%) cultureconfirmed tuberculosis cases, including 4/5 (80%) HIV-infected and 4/12 (33%) HIV-uninfected children. Sputum Xpert detected 11/17 (65%) cases. Stool holds promise for PTB diagnosis in HIV-infected children.

The diagnosis of pulmonary tuberculosis (PTB) remains challenging in young children because they seldom expectorate spontaneously, making it difficult to obtain a representative specimen from the lower respiratory tract, and because PTB is typically paucibacillary. As a result, microbiological confirmation is less frequently achieved than in adult cases [1]. Culture confirmation of disease can take weeks and disease progresses rapidly in young children. Consequently, rapid diagnostic methods such as Xpert MTB/RIF (Xpert) are an important advance.

The accuracy of Xpert testing of induced sputum (IS) [2], nasopharyngeal aspirates [3], and gastric lavage aspirates [4] from children has recently been reported. However, obtaining such specimens, especially in primary care settings, can be difficult. In contrast, it is relatively easy to obtain stool samples. Since young children frequently swallow their sputum, *Mycobacterium tuberculosis* may be detected in the stool of children with PTB [5]. We therefore performed a pilot study of the diagnostic accuracy of Xpert testing of stool samples from children with suspected PTB.

## MATERIALS AND METHODS

### Study Design, Setting, and Population

This was a prospective study in which samples were obtained from an ongoing cohort based at a primary care clinic (Nolungile Clinic, Khayelitsha, South Africa) and a tertiary pediatric hospital (Red Cross Children's Hospital, Cape Town, South Africa). Children (age <15 years) presenting with suspected PTB were enrolled consecutively (from 11 July 2011 to 26 March 2012). Criteria for enrollment were a cough lasting longer than 2 weeks and at least 1 of the following: (1) household tuberculosis contact in the prior 3 months, (2) weight loss or failure to gain weight in the previous 3 months, (3) a positive tuberculin skin test, or (4) a chest radiograph suggestive of PTB. Children were evaluated at 3 months to assess recovery or response to treatment. We included all children from whom both stool and sputum samples had been collected and where >1.5 g of stool was available (only 0.15 g was used for testing).

Children were excluded if they had received treatment for tuberculosis lasting longer than 72 hours, they did not live in Cape Town, they were unable to attend follow-up visits, informed consent was not given, or an IS sample was not obtained. Written informed consent was obtained from a parent or legal guardian. The Research Ethics Committee of the Faculty of Health Sciences, University of Cape Town, approved the study.

### Procedures

Routine history and physical examination were performed at enrollment. All children received baseline chest radiography and human immunodeficiency virus (HIV) testing (HIV rapid test followed by confirmatory polymerase chain reaction for children age <18 months). Patient stool (a single convenience specimen) and IS (2 specimens) were collected at baseline as previously described [6]. IS specimens were processed within 2 hours. Stool specimens were stored at −80°C within 2 hours; Xpert testing was performed within 6 months of storage. Classification of tuberculosis status was as follows: “definite tuberculosis,” children culture-positive for *M. tuberculosis*; “not tuberculosis,” children culture-negative for *M. tuberculosis* who were not started on tuberculosis treatment and clinically improved at the 3 month follow-up visit; and “possible tuberculosis,” all other children.

***Sputum Processing***

Samples were decontaminated with N-acetyl-L-cysteine and 1% sodium hydroxide (final concentration) and then concentrated by centrifugation. Pellet was resuspended in 1.5 mL phosphate buffered saline (PBS). BACTEC MGIT (Becton Dickinson) culture was performed using a 0.5-mL aliquot of the resuspended sample and Xpert using 0.7 mL of the resuspended sample, to which 1.4 mL of Xpert reagent was added and processed per the manufacturer's instructions.

***Stool Processing***

Next, 0.15 g of thawed stool (confirmed by weighing) was retrieved using pediatric FLOQSwabs (Copan Italia, Brescia, Italy). Swabs were then placed in 2.4 mL PBS and vortexed briefly before being removed. The sample was left undisturbed for 20 minutes at room temperature to allow large particles to settle before 2 aliquots of 1-mL supernatant were removed. One aliquot was tested immediately with Xpert and the other was stored at 4°C for later duplicate testing (within 1 week). Prior to Xpert testing, the sample was centrifuged at 3200 g for 15 minutes. The supernatant was discarded and pellet was resuspended in 1 mL PBS. Xpert testing was then performed per the manufacturer's instructions using a 2:1 ratio of Xpert reagent to sample.

### Statistical Analysis

The reference standard was a positive liquid culture from at least 1 IS sample. Statistical analysis was performed with Stata version 11.0 (StataCorp, College Station, Texas). Diagnostic test characteristics were determined with 95% confidence intervals (CIs). A 2-sample test of proportion was used to compare the sensitivity of Xpert in HIV-infected and -uninfected children.

## RESULTS

Testing was performed on 115 children, median age 31 months (interquartile range 19–57 months) of whom 17 (14.8%) were HIV infected and 67 (58.3%) were hospitalized. Of the 115 children, 17 (14.8%) were classified as having definite PTB, 48 (41.7%) as possibly having tuberculosis, and 50 (43.5%) as not having tuberculosis. There were no cases of rifampicin resistance detected.

Duplicate Xpert testing of a single stool sample detected 8/17 (47.1%) children with definite tuberculosis (each Xpert test detected 7/17 cases, 41.2%; Table 1). One child with possible tuberculosis was positive by stool Xpert testing but negative by culture and IS Xpert testing. This child was treated for tuberculosis, and clinical improvement was documented at follow-up. No children in the not tuberculosis group were positive by stool Xpert testing. The median age of children with definite tuberculosis who had a positive stool Xpert result (87 months; 95% CI, 38–127) was not significantly higher than that of children with a negative result (30 months; 95% CI, 24–55; *P* = .102). Children with definite tuberculosis who had a positive stool Xpert test had a similar incidence of alveolar consolidation (100% vs 63%, *P* = .20) and nodal compression (86% vs 100%, *P* = .47) on chest radiography as children with a negative Xpert test.

**Table 1.** Diagnostic Accuracy of Xpert (On Stool and Induced Sputum) and Smear Microscopy (On Induced Sputum), When Using Liquid Culture of 2 Induced Sputum Samples as a Reference Standard

Diagnostic Test	Sensitivity, Test Positive/Total Culture Positive, % (95% CI)	Specificity, Test Negative/Total Culture Negative, % (95%CI)
Stool Xpert^a^		
All	8/17, 47.1% (26.2–69.0)	97/98, 99.0% (94.4–99.8)
HIV infected	4/5, 80.0% (37.6–96.4)	12/12, 100% (75.8–100)
HIV uninfected	4/12, 33.3% (13.8–61.0)	85/86, 98.8% (93.7–99.8)
Induced sputum Xpert		
All	11/17, 64.7% (41.3–86.7)	95/98, 96.9% (91.4–99.0)
HIV infected	3/5, 60.0% (23.1–88.2)	11/12, 91.7% (64.6–98.5)
HIV uninfected	8/12, 66.7% (39.1–86.2)	85/86, 98.8% (93.7–99.8)
Induced Sputum Smear		
All	4/17, 23.5% (9.6–47.3)	98/98, 100% (96.2–100)
HIV infected	3/5, 60.0% (23.1–88.2)	12/12, 100% (75.8–100)
HIV uninfected	1/12, 8.3% (1.5–35.4)	86/86, 100% (95.7–100)

Abbreviations: CI, confidence interval; HIV, human immunodeficiency virus.

^a^ Two Xpert tests performed on a single stool sample.

Xpert testing of IS detected 11/17 (64.7%) definite tuberculosis cases, while 3 children in the possible tuberculosis group were positive by IS (but not stool) Xpert testing (all of whom were treated for tuberculosis and had clinical improvement documented at the 3-month follow-up visit). No children in the not tuberculosis group were positive by IS Xpert testing. The sensitivity of Xpert on stool versus IS was not significantly different (*P* = .30); however, this analysis is limited by the small sample size (Table 2).

**Table 2.** Comparison of Stool and Induced Sputum Xpert Test Results

	Stool Xpert		
Diagnostic Test	Positive	Negative	Total
Induced sputum Xpert			
Positive	7	6	13
Negative	2	99	101
Total	9	105	114^a^

^a^One Xpert result on induced sputum recorded as indeterminate.

Xpert testing on stool samples was positive in 4/5 (80%) HIV-infected children with definite tuberculosis versus 4/12 (33.3%) HIV-uninfected children with definite tuberculosis (*P* = .08 for comparison of HIV-infected and -uninfected children). There were no significant differences in chest radiography between HIV-infected and -uninfected children with culture-confirmed tuberculosis. All HIV-infected children had alveolar consolidation and nodal compression. Of the HIV-uninfected children, 72% had alveolar consolidation and 91% had nodal compression (*P* = .51 for alveolar consolidation and *P* = 1.0 for nodal compression). No children with culture confirmed tuberculosis had military infiltrates on chest radiography.

Smear microscopy of IS detected 4/17 cases (23.5%) of definite tuberculosis. Among children with definite tuberculosis, 3/5 (60%) HIV-infected children were smear positive compared with 1/12 (8%) HIV-uninfected children (*P* = .09). All 4 smear-positive cases were detected by both stool and sputum Xpert testing.

## DISCUSSION

This pilot study suggests that Xpert testing of stool samples from children with suspected PTB holds promise as a diagnostic approach, particularly for HIV-infected children in whom duplicate testing of a single stool sample detected 4/5 cases. The number of children included in this study was too small to evaluate the relative performance of Xpert on stool compared with IS.

Results from stool Xpert testing in a recent pilot study of 17 children (14 of whom received Xpert testing of both stool and gastric aspirate and 3 received Xpert testing of stool only) [7] showed that stool Xpert testing (test was performed on decontaminated stool sediment, not directly on stool) detected 3 of 4 cases of culture-confirmed intrathoracic tuberculosis and 3 of 6 cases where *M. tuberculosis* was cultured from any site.

Our study used small volumes of stool for testing, as use of larger volumes led to failed Xpert assays (data not shown), possibly due to clogging of filters in the Xpert cartridge. Optimized protocols, which are currently being developed for Xpert testing of stool samples, will permit testing of significantly larger volumes of stool and are likely to enhance sensitivity. Further studies of larger numbers of children utilizing these optimized specimen-processing protocols are required.

Xpert testing has enabled rapid diagnosis of PTB and detection of rifampicin resistance in children, which promotes timely initiation of appropriate therapy. Because Xpert is designed for use close to the point of care, use of stool as a sample for Xpert testing may be especially appropriate, given the ease with which stool is obtained in primary care settings. The finding that suggests relatively high sensitivity in HIV-infected children is important, as this group of children is at high risk for rapid progression of disease and mortality. Thus Xpert testing on stool may identify a group of children in whom rapid initiation of tuberculosis therapy may be life saving.
