# Nicol MP et al (Clin Infect Dis 2013; 57:e18–21)

**DOI:** 10.1093/cid/ciu347

**Published:** 2014-07-01

An error appeared in 1 August 2013 issue of the journal [Nicol MP, Spiers K, Workman L, et al. Xpert MTB/RIF Testing of Stool Samples for the Diagnosis of Pulmonary Tuberculosis in Children. Clin Infect Dis **2013**; 57:e18–21]. The access control for the article was CC-BY-NC-ND, and has since been changed to CC-BY. The copyright statement now reads as follows:

© The Author 2014. Published by Oxford University Press on behalf of the Infectious Diseases Society of America. This is an Open Access article distributed under the terms of the Creative Commons Attribution License (http://creativecommons.org/licenses/by/3.0/), which permits unrestricted reuse, distribution, and reproduction in any medium, provided the original work is properly cited.

The authors regret this error.

